# Giant Axonal Neuropathy: A Case Report of Subclinical Childhood Manifestations

**DOI:** 10.7759/cureus.54368

**Published:** 2024-02-17

**Authors:** Ahmed K Bamaga, Osama Y Muthaffar, Anas S Alyazidi, Rakan Abu Alqam

**Affiliations:** 1 Pediatrics, King Abdulaziz University Hospital, Jeddah, SAU; 2 Pediatrics, King Abdulaziz University Faculty of Medicine, Jeddah, SAU; 3 Medicine, King Abdulaziz University Faculty of Medicine, Jeddah, SAU

**Keywords:** cns involvement, hereditary neuropathy, gigaxonin mutations, giant axons, giant axonal neuropathy

## Abstract

Giant axonal neuropathy (GAN) is a rare, inherited neurodegenerative disease that affects both the central and peripheral nervous systems. It is mostly characterized by a progressive loss of motor and sensory function, which can begin in early childhood. GAN is thought to be caused by a mutation in the GAN gene on chromosome 16q24.1. We report a seven-year-old Saudi male child with GAN who was diagnosed using whole-exome sequencing. The child presented with a history of progressive weakness and muscle wasting in the arms and legs as well as difficulty walking. The sequencing identified a mutation in the GAN gene (NM_022041.3: c.1456G>A). Electrodiagnostic studies showed evidence of diffuse axonal motor and sensory polyneuropathy involving cranial nerves. This case report adds to the growing evidence that whole-exome sequencing can be a useful tool for diagnosing rare inherited neuromuscular disorders. It also highlights the importance of early diagnosis and intervention for this condition.

## Introduction

Giant axonal neuropathy (GAN) is a rare progressive hereditary autosomal recessive disorder that affects both the central and peripheral nervous systems. It is characterized by the presence of oversized axons [1,2]. The genetic mutation responsible for GAN can be located on chromosome 16q24.1 within the GAN gene [3]. Initial clinical symptoms typically appear around the age of four, progressing in severity throughout the early 20s and often leading to premature death in the second or third decade of life [1,4]. This condition results in sensorimotor polyneuropathy, which manifests in various neurological abnormalities including distal muscle weakness, muscle atrophy, sensory deficits, loss of deep tendon reflexes, and difficulties with walking and maintaining posture [1,3]. Additionally, individuals with GAN may also experience oculomotor and facial cranial nerve dysfunction [1,4]. As the disease progresses, it can involve regions of the brain including the cerebral cortex, cerebellum, brain stem, and pyramidal tract, leading to symptoms like dysarthria, intellectual impairment, seizures, and cerebellar dysfunction [5]. One of the diagnostic indicators of GAN is the presence of coarse, kinky hair, although this symptom may not always be present. The specific factors contributing to the various clinical presentations of GAN are not fully elucidated, and differential diagnosis should consider conditions like classic infantile neuroaxonal dystrophy (INAD), Charcot-Marie-Tooth hereditary neuropathy type 4 (CMT4), and leukodystrophies, including arylsulfatase-A deficiency [3]. In this case report, we present the clinical details of a seven-year-old child diagnosed with GAN, who exhibited electrodiagnostic evidence of diffuse axonal motor and sensory polyneuropathy affecting the cranial nerves.

## Case presentation

This patient is a seven-year-old Saudi male who presented with progressive gait disturbance, frequent falls, and facial diplegia. He was one of three children in the family. Both parents are heterozygous carriers of a pathogenic variant in the GAN gene with positive first-degree consanguinity. They are otherwise a healthy couple with no neurological disorders or hereditary diseases in the family. The patient was a product of full-term uneventful pregnancy and neonatal history, delivered through spontaneous vaginal delivery (SVD). At the age of 18 months, he started to walk while experiencing frequent falls. His walking difficulties became progressive over time. He is still able to walk, although he has bilateral foot drop. His nerve conduction studies including facial nerve showed evidence of diffuse axonal motor and sensory polyneuropathy.

Electromyography (EMG) revealed evidence of chronic denervation with no evidence of re-innervation. He had no history of seizures. His school performance is excellent with no learning difficulties. On physical examination, he had no dysmorphic features; however, he had kinky thick hair, horizontal nystagmus, facial weakness, and distal more than proximal weakness in both upper and lower limbs with reduced deep tendon reflexes. He has a positive Romberg sign, moderate speech delay, and a high steppage gate. The audiogram and ocular assessments were both normal. His laboratory workup included a complete blood count (CBC), metabolic profile, blood glucose, lactate, ammonia, renal function tests, vitamin B12, creatinine kinase, and thyroid hormones, all of which were within normal levels. Brain magnetic resonance imaging (MRI) was conducted on the brain when the patient was six years old, and the results were normal.

As for genetic testing, following appropriate ethical and logistical measures, we obtained the genetic sample of the patients. Whole-exome sequencing (WES) testing was carried out on the two unaffected siblings and their respective parents. DNA capture probes using next-generation sequencing (NGS)-based copy number variation (CNV) analysis with Illumina array were performed. We identified a novel (NM_022041.3: c.1456G>A) mutation in the GAN gene. Subsequently, the patient was diagnosed with GAN type 1. The patient is currently enrolled in regular physical and occupational therapy sessions.

## Discussion

**Figure 1:**
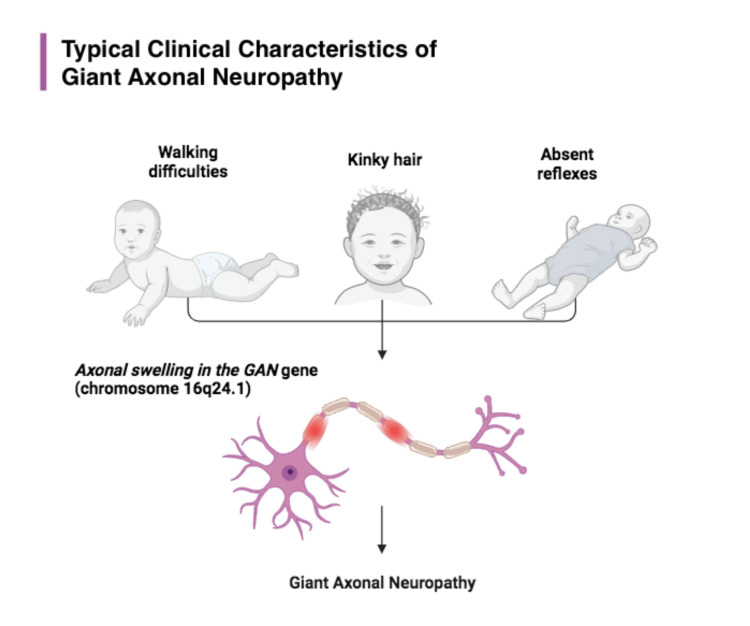
Typical clinical characteristics of patients with GAN GAN: Giant axonal neuropathy. Source: This figure is an original work created by the authors of the study.

To date, approximately 50 families have been identified with biallelic GAN pathogenic variants [6]. Among these cases, a broad phenotypic spectrum was reported with varied severities [7]. Despite this, the predictive factors of the disease remain unknown, and previous studies that investigated the involvement of 47 mutated alleles in the GAN gene are yet to establish a definitive genotypic-phenotypic correlation [8]. Classical features including kinky hair, frequent falls, and sensory-motor polyneuropathy are frequently associated with the disorder (Figure 1).

In a recent study that included three patients from two unrelated families, the previously mentioned features, in addition to walking difficulties and ataxic gait, were reported [9]. Similarly, our patient presented with the same features. One could predict a similar genotypic pattern of the cases; however, in the reported cases, a novel nonsense variant (NM_022041.3:c.1162del [p.Leu388Ter]) and missense variant (NM_022041.3:c.370T>A [p.Phe124Ile]) were reported. In our case, we identified a different novel variant (NM_022041.3:c.1456G>C). Despite such genetic disparities, all three mutations manifested with similar clinical presentation. Furthermore, the neurologic examination is often expected to prevail absent lower extremity reflexes, walking difficulties, ataxic gait, or kinky hair; however, spasticity, paresis, muscular atrophy or fasciculation, myoclonus, stiffness, or dystonic pattern can usually be unremarkable [9]. This is confirmed by studies that reviewed over 63 reported cases of the GAN variant [9]. These findings were echoed by Guo et al. [10] who also investigated 55 cases of the GAN variant showing clinical features that are compatible with the clinical presentation of our cases. Moreover, determining the severity and prognosis of patients remains an active area of research.

In some investigations, the classical presentation of kinky hair was presented as potentially an early sign of the disorder that presents before other manifestations [11]. Furthermore, another study demonstrated that the early appearance of kinky hair could be related to severe phenotype [12]. With all those clinical hallmarks, it is still arguable to rely merely on clinical manifestations. This has been also suggested based on studies that analyzed the features of 75 children with peripheral inherited neuropathies [13]. For that, it is widely recommended to use the aid of neuropathology findings in nerve biopsy as well as laboratory evaluation and other neuroradiological modalities to evaluate, monitor, and follow up with cases carrying this variant [14,15]. As such, this was the adopted approach in our case that yielded successful early detection of the variant and subsequently proper follow-up plans.

## Conclusions

A pathogenic variation within the GAN gene can manifest in distinct clinical ways. Therefore, it is crucial to consider genetic and laboratory testing, particularly when encountering potential carriers of the mutation or when clinical suspicion arises, especially in cases with milder phenotypes. The study identified several consistent features associated with the GAN gene variation, without any atypical presentations, thus confirming these characteristics as potential indicators of the condition. These findings reflect the varied observations in patients with the GAN variation. This study's results contribute to advance our knowledge of this complex disorder. Research that focuses on the clinical presentation of rare diseases with limited reported cases in the medical literature can help healthcare professionals achieve accurate diagnoses.
